# Temporal trends in non-small cell lung cancer survival in Sweden

**DOI:** 10.1038/sj.bjc.6603591

**Published:** 2007-01-23

**Authors:** D R Brooks, Å Klint, P W Dickman, E Ståhle, M Lambe

**Affiliations:** 1 Department of Epidemiology, Boston University School of Public Health, 715 Albany Street, Boston, MA 02118, USA; 2 Department of Medical Epidemiology and Biostatistics, Karolinska Institutet, PO Box 281, SE-171 77, Stockholm, Sweden; 3 Department of Thoracic Surgery, University Hospital, SE-75185, Uppsala, Sweden

**Keywords:** lung cancer, adenocarcinoma, squamous cell carcinoma, survival, Sweden

## Abstract

We modeled temporal trends in the 1- and 5-year survival of 32 499 patients with adenocarcinoma and squamous cell carcinoma of the lung in the Swedish Cancer Register between 1961 and 2000. The 1-year relative survival for adenocarcinoma improved from 37% for patients diagnosed 1961–1965 to 45% for those diagnosed 1996–2000 and from 39 to 45% for squamous cell carcinoma. The adjusted excess mortality ratios for the period 1996–2000 compared with 1961–1965 were 0.80 for adenocarcinoma and 0.81 for squamous cell carcinoma. Thus, a previous report in a Dutch study of a relatively worsening prognosis for adenocarcinoma over time could not be confirmed.

Over the past three decades in many Western countries, there has been a relative increase in the incidence of adenocarcinoma of the lung compared with other histological types (Janssen-Heijnen and Coebergh, 2001; 2003). The same phenomenon has been observed in Sweden, particularly among men (Myrdal *et al*, 2001). This shift cannot be attributed simply to changes in classification or diagnostic methods (Charloux *et al*, 1997). It is more likely to reflect the increased use of lower tar and nicotine cigarettes, which are suspected of preferably favouring the development of adenocarcinoma (Wynder and Muscat, 1995; Stellman *et al*, 1997; Thun *et al*, 1997).

Data from a regional population-based cancer register in the Netherlands during the period 1975–1994 indicated not only doubling of the relative incidence of adenocarcinoma but also a decline in its relative survival, even as relative survival ratios (RSRs) remained unchanged for other forms of non-small cell lung cancer. It was hypothesised that the decreased survival was also related to increased use of lower-yield cigarettes, and suggested that among adenocarcinomas, those caused by smoking may be more aggressive than those unrelated to smoking (Janssen-Heijnen *et al*, 2001).

We used nationwide, population-based data for Sweden over a 40-year period to investigate survival trends for adenocarcinoma compared with squamous cell carcinoma, the other main type of non-small cell lung cancer.

## METHODS

Data for this analysis were derived from the national Swedish Cancer Register (SCR), which was established in 1958. Swedish law mandates the report of all newly diagnosed malignant tumours, as well as benign tumours from selected sites. The completeness of reporting to the SCR has been very high throughout its existence; in 1975, it was estimated that almost 100% of all malignant tumours were reported (National Board of Health and Welfare, 1980). The SCR uses the ICD-7 to classify primary site and the PAD code, assigned by the diagnosing pathologist, to denote the histological subtype. Approximately 97% of the cases are morphologically verified with little change over time (National Board of Health and Welfare, 1998).

Criteria for inclusion in this analysis included age 20–80, diagnosis of a malignant lung tumour (ICD-7: 162.1) between 1961 and 2000 and no prior diagnosis of cancer. Because large cell carcinoma and small cell lung cancer were combined as one category until 1985, we limited the analyses to individuals with the two main types of non-small cell lung cancer: adenocarcinoma (PAD: 96) and squamous cell carcinoma (PAD: 146). From the 39 714 cases originally eligible, we excluded 7 215 individuals who were diagnosed at autopsy or survived less than 1 month after diagnosis. The final dataset consisted of 32 499 patients.

Follow-up for mortality is conducted routinely by the SCR through linkage with the Cause of Death Register. We included follow-up for death through the end of 2002 and calculated 1- and 5-year RSRs by sex and histologic type for individuals diagnosed during successive 5-year calendar periods (1961–1965, 1966–1970,…, 1996–2000). The RSR represents the ratio of observed survival among individuals with lung cancer to expected survival based on the sex, age (5-year categories) and period-specific mortality of the general population (Ederer *et al*, 1961). Data for expected survival were derived from population life tables.

To study changes in survival over calendar period of diagnosis for adenocarcinoma and squamous cell carcinoma while adjusting for age and sex, we modelled the excess mortality (observed minus expected) using Poisson regression. The excess mortality ratio represents the relative excess mortality within each 5-year calendar period compared with the reference period of 1961–1965 (Dickman *et al*, 2004). We calculated 95% confidence intervals for both RSRs and excess mortality ratios. Analyses were performed using Stata (Dickman *et al*, 2007). The study was approved by the Regional Ethics Board of Akademiska Sjukhuset, Uppsala.

## RESULTS

Table 1 provides information on the number of cases of lung adenocarcinoma and squamous cell carcinoma by sex and decade. The proportion of all cases occurring among women doubled from 17% during 1961–1970 to 36% during 1991–2000. Adenocarcinoma steadily increased from 17% of all cases among men during 1961–1970 to 41% in 1991–2000. Among women, adenocarcinoma accounted for 60% of cases during 1961–1990 and increased to 65% during the most recent period under study (1991–2000).

The 1-year RSR for adenocarcinoma improved from 37% for diagnoses in 1961–65 to 45% for 1996–2000, and from 38% to 45% for squamous cell carcinoma (Table 2). The corresponding figures for the 5-year RSR were 14% (1961–1965) to 17% (1996–2000) for both adenocarcinoma and squamous cell carcinoma. Although absolute estimates of relative survival decreased in a separate analysis that included patients dying within 1 month of diagnosis, the survival patterns remained essentially unchanged (data not shown). Even over the specific period (1975–1994) covered by the previous report (Janssen-Heijnen *et al*, 1998), both 1- and 5-year RSRs remained essentially unchanged in our data for both histologic groups.

The excess mortality ratios, adjusted for gender and age, for the period 1996–2000 compared with 1961–1965 were 0.80 for adenocarcinoma and 0.81 for squamous cell carcinoma (Table 2). For both histologic groups, a substantial fraction of the improvement occurred during the most recent calendar period.

## DISCUSSION

Over the past 40 years, there has been modest improvement in 1-year and little change in 5-year relative survival for lung adenocarcinoma and squamous cell carcinoma in Sweden, most of the improvement being in the last decade, perhaps reflecting more intensive early detection, the introduction of new cytotoxic regimens and implementation of guidelines promoting chemotherapy in advanced disease (American Society of Clinical Oncology, 1997).

Our finding that trends in survival over time were similar for both cell types did not confirm the earlier observation (Janssen-Heijnen *et al*, 1998) of a worsening trend with adenocarcinoma. Reasons for the different results in our studies are not obvious as both were based on population-based registers with similar eligibility criteria and analytical methods. Historically, both smoking prevalence and lung cancer mortality have been substantially higher in the Netherlands than Sweden (Statistics Sweden, 1997; Liaw *et al*, 2005). Consequently, the proportion of adenocarcinomas attributable to smoking was probably greater in the Netherlands, and this may have contributed to the survival differences. In addition, we cannot rule out differences in diagnostic, treatment or pathological classification practices between the two countries.

The increased incidence of adenocarcinoma observed in Sweden has also occurred on a global scale (Charloux *et al*, 1997; Jansssen-Heijnen and Coebergh, 2001). Originally, adenocarcinoma was considered to occur primarily among non-smokers (Doll *et al*, 1957; Kreyberg, 1961), but later studies demonstrated that it is also strongly associated with smoking (Thun *et al*, 1997; Simonato *et al*, 2001; Yang *et al*, 2002). The increased incidence of adenocarcinoma among smokers may be explained by the widespread introduction of filter and lower-tar and nicotine cigarettes (Wynder and Muscat, 1995; Stellman *et al*, 1997; Thun *et al*, 1997).

In 1946, 50% of men and 9% of women in Sweden were daily smokers (www.tobaksfakta.org/default.aspx?id=3622). Filter cigarettes first became available in Sweden during the late 1950s, whereas reduced tar and nicotine cigarettes were introduced in the early 1970s and now dominate the market. The substantial increase in adenocarcinoma as a proportion of all cases among men over the past 40 years is consistent with early use of non-filter cigarettes followed by a transition to filter and low-tar cigarettes. Because of their later uptake of smoking, women were always more likely to smoke filter and low-tar cigarettes. Adenocarcinoma has always occurred more frequently than squamous cell carcinoma among women. However, cases in the earlier period were more likely to be non-smoking-related, whereas more recent cases are more likely to be caused by smoking.

Strengths of our study included the nationwide scope, the large number of cases over a 4-decade period and the high case ascertainment. Throughout the study period, less than 3% of all cases lacked histological classification. However, we lacked information on stage at diagnosis and were unable to evaluate the trend in the stage distribution by histologic type. Janssen-Heijnen *et al* (1998) found that the proportion of cases diagnosed at a localised stage decreased over the study period for adenocarcinoma but increased for squamous cell carcinoma. We also had no information on smoking, which would have permitted a direct comparison of the survival of persons diagnosed with adenocarcinoma according to their smoking history. One registry-based study in Japan with such information found smoking was associated with poorer survival for adenocarcinoma but not for other histological types (Kato *et al*, 1990).

In conclusion, we did not find a worsening trend for survival for adenocarcinoma of the lung in Sweden over a 40-year period. Nevertheless, a variety of studies suggests that adenocarcinoma due to smoking differs from other adenocarcinomas (Ahrendt *et al*, 2001; Ishikawa *et al*, 2002; Miura *et al*, 2002; Toyooka *et al*, 2006). It is too soon to decide whether or not these differences have implications for patient survival. Additional studies with information on tobacco use would help determine whether adenocarcinoma has become more deadly as it has become more common and more closely associated with cigarette smoking.

## Figures and Tables

**Table 1:** Number of lung cancer cases (adenocarcinoma or squamous cell carcinoma only), by calendar period and gender, Swedish Cancer Register, 1961–2000

	**1961–1970**		**1971–1980**		**1981–1990**		**1991–2000**	
	**M**	**F**	**M**	**F**	**M**	**F**	**M**	**F**
Overall	4084 (83)	864 (17)	6295 (81)	1451 (19)	6987 (74)	2428 (26)	6605 (64)	3785 (36)

Age at diagnosis								
20–40	47 (1)	31 (4)	53 (<1)	30 (2)	77 (1)	64 (3)	40 (<1)	55 (1)
41–50	263 (6)	95 (11)	323 (5)	140 (10)	380 (5)	257 (11)	360 (5)	396 (10)
51–60	1047 (26)	214 (25)	1368 (22)	334 (23)	1275 (18)	523 (22)	1146 (17)	915 (24)
61–70	1843 (45)	288 (33)	2641 (42)	553 (38)	2885 (41)	921 (38)	2559 (39)	1264 (33)
71–80	884 (22)	236 (27)	1910 (30)	394 (27)	2370 (34)	663 (27)	2500 (38)	1155 (31)

Histologic group								
Adenocarcinoma	688 (17)	516 (60)	1290 (20)	871 (60)	2153 (31)	1447 (60)	2693 (41)	2473 (65)
Squamous cell carcinoma	3396 (83)	348 (40)	5005 (80)	580 (40)	4834 (69)	981 (40)	3912 (59)	1312 (35)

Percentage within each decade in parentheses.

**Table 2:** One- and 5-year RSRs and excess mortality ratios with 95% CI over calendar period by gender and histologic group, Swedish Cancer Register, 1961–2000

	**1961–1965**	**1966–1970**	**1971–1975**	**1976–1980**	**1981–1985**	**1986–1990**	**1991–1995**	**1996–2000**
	**% (95% CI)**	**% (95% CI)**	**% (95% CI)**	**% (95% CI)**	**% (95% CI)**	**% (95% CI)**	**% (95% CI)**	**% (95% CI)**
1-year RSR								
Overall								
Adenocarcinoma	37.0 (32.8–41.3)	32.6 (29.1–36.2)	36.9 (33.8–40.0)	40.0 (37.2–42.8)	38.0 (35.6–40.4)	37.7 (35.5–39.9)	39.6 (37.6–41.6)	44.7 (42.8–46.6)
Squamous cell carcinoma	38.5 (36.1–40.9)	40.3 (38.2–42.5)	40.7 (38.8–42.7)	42.8 (40.9–44.6)	44.2 (42.4–46.0)	41.4 (39.5–43.3)	41.6 (39.7–43.4)	45.3 (43.2–47.3)

Male								
Adenocarcinoma	32.2 (26.8–37.8)	30.0 (25.5–34.6)	36.5 (32.5–40.5)	36.5 (32.9–40.1)	34.1 (31.1–37.1)	36.6 (33.8–39.4)	37.3 (34.6–40.0)	41.7 (39.0–44.3)
Squamous cell carcinoma	39.0 (36.5–41.5)	40.8 (38.5–43.1)	41.0 (38.9–43.0)	43.3 (41.3–45.2)	45.1 (43.2–47.1)	41.4 (39.3–43.5)	41.6 (39.4–43.7)	44.4 (42.0–46.9)

Female								
Adenocarcinoma	43.1 (36.5–49.6)	36.1 (30.6–41.7)	37.6 (32.5–42.6)	44.9 (40.5–49.3)	43.9 (40.0–47.8)	39.3 (35.9–42.7)	42.1 (39.2–45.0)	47.8 (45.1–50.5)
Squamous cell carcinoma	33.8 (26.4–41.4)	35.8 (29.0–42.7)	38.0 (31.7–44.3)	38.7 (33.5–43.9)	39.3 (35.0–43.6)	41.3 (36.8–45.7)	41.6 (37.7–45.5)	47.3 (43.4–51.1)

5-year RSR								
Overall								
Adenocarcinoma	13.8 (10.9–17.2)	10.9 (8.6–13.6)	12.9 (10.8–15.3)	13.8 (11.9–16.0)	12.5 (10.9–14.3)	12.9 (11.3–14.5)	14.5 (13.0–16.0)	16.7 (15.1–18.4)
Squamous cell carcinoma	14.2 (12.5–16.1)	13.6 (12.0–15.2)	14.5 (13.0–16.0)	13.9 (12.5–15.2)	13.9 (12.6–15.3)	14.1 (12.7–15.5)	14.9 (13.5–16.3)	17.1 (15.3–18.9)

Male								
Adenocarcinoma	12.9 (9.1–17.3)	9.3 (6.6–12.6)	13.1 (10.3–16.2)	11.4 (9.0–14.1)	9.9 (8.0–12.1)	12.5 (10.6–14.7)	11.3 (9.5–13.2)	13.4 (11.3–15.7)
Squamous cell carcinoma	14.3 (12.4–16.2)	13.4 (11.8–15.1)	14.8 (13.3–16.4)	14.0 (12.6–15.5)	14.1 (12.7–15.6)	13.9 (12.4–15.5)	13.9 (12.4–15.5)	16.9 (14.8–19.1)

Female								
Adenocarcinoma	15.1 (10.6–20.4)	13.2 (9.4–17.6)	12.8 (9.5–16.6)	17.3 (14.0–20.9)	16.3 (13.5–19.4)	13.4 (11.0–15.9)	17.9 (15.7–20.3)	20.2 (17.7–22.7)
Squamous cell carcinoma	13.7 (8.6–20.1)	15.2 (10.2–21.0)	11.0 (7.3–15.6)	12.7 (9.3–16.6)	12.9 (10.0–16.2)	14.8 (11.6–18.3)	18.1 (15.1–21.4)	17.6 (14.4–21.2)

Excess mortality ratio								
Overall								
Adenocarcinoma	1 (ref)	1.13 (1.00–1.28)	0.98 (0.87–1.10)	0.91 (0.82–1.02)	0.94 (0.85–1.05)	0.94 (0.85–1.05)	0.90 (0.81–1.00)	0.80 (0.72–0.88)
Squamous cell carcinoma	1 (ref)	0 96 (0.89–1.03)	0.93 (0.87–1.00)	0.90 (0.84–0.96)	0.87 (0.82–0.93)	0.91 (0.85–0.97)	0.89 (0.83–0.95)	0.81 (0.76–0.87)

Male								
Adenocarcinoma	1 (ref)	1.13 (0.96–1.33)	0.92 (0.79–1.08)	0.93 (0.80–1.08)	0.97 (0.84–1.12)	0.88 (0.77–1.02)	0.90 (0.78–1.03)	0.79 (0.69–0.91)
Squamous cell carcinoma	1 (ref)	0.96 (0.89–1.04)	0.92 (0.86–0.99)	0.90 (0.83–0.96)	0.86 (0.80–0.92)	0.91 (0.84–0.98)	0.90 (0.84–0.97)	0.81 (0.75–0.88)

Female								
Adenocarcinoma	1 (ref)	1.12 (0.93–1.36)	1.06 (0.88–1.27)	0.89 (0.74–1.06)	0.90 (0.76–1.07)	1.02 (0.87–1.20)	0.91 (0.78–1.07)	0.81 (0.69–0.95)
Squamous cell carcinoma	1 (ref)	0.92 (0.73–1.16)	0.98 (0.79–1.23)	0.90 (0.73–1.11)	0.87 (0.71–1.06)	0.82 (0.67–1.00)	0.75 (0.62–0.91)	0.71 (0.58–0.86)

Abbreviations: CI=confidence interval; RSR=relative survival ratio.

Estimates are conditional on survival at least 1 month and are adjusted for age.
